# The limitations of employment as a tool for social inclusion

**DOI:** 10.1186/1471-2458-10-621

**Published:** 2010-10-19

**Authors:** Liana S Leach, Peter Butterworth, Lyndall Strazdins, Bryan Rodgers, Dorothy H Broom, Sarah C Olesen

**Affiliations:** 1 Centre for Mental Health Research, The Australian National University, Canberra, 0200, Australia; 2 National Centre for Epidemiology and Population Health, The Australian National University, Canberra, 0200, Australia; 3 Australian Demographic and Social Research Institute, The Australian National University, Canberra, 0200, Australia

## Abstract

**Background:**

One important component of social inclusion is the improvement of well-being through encouraging participation in employment and work life. However, the ways that employment contributes to wellbeing are complex. This study investigates how poor health status might act as a barrier to gaining good quality work, and how good quality work is an important pre-requisite for positive health outcomes.

**Methods:**

This study uses data from the PATH Through Life Project, analysing baseline and follow-up data on employment status, psychosocial job quality, and mental and physical health status from 4261 people in the Canberra and Queanbeyan region of south-eastern Australia. Longitudinal analyses conducted across the two time points investigated patterns of change in employment circumstances and associated changes in physical and mental health status.

**Results:**

Those who were unemployed and those in poor quality jobs (characterised by insecurity, low marketability and job strain) were more likely to remain in these circumstances than to move to better working conditions. Poor quality jobs were associated with poorer physical and mental health status than better quality work, with the health of those in the poorest quality jobs comparable to that of the unemployed. For those who were unemployed at baseline, pre-existing health status predicted employment transition. Those respondents who moved from unemployment into poor quality work experienced an increase in depressive symptoms compared to those who moved into good quality work.

**Conclusions:**

This evidence underlines the difficulty of moving from unemployment into good quality work and highlights the need for social inclusion policies to consider people's pre-existing health conditions and promote job quality.

## Background

Participation in employment is often an element of social inclusion policy designed to improve health and well-being. However, the reciprocal relationships between employment and health are complex. On the one hand, health conditions affect the likelihood of gaining employment, while on the other, unemployment has a negative health impact (particularly on mental health) [1]. Additionally, not all jobs are beneficial to health. Indeed, jobs with poor working conditions can erode health and well-being. This paper investigates the interplay between employment, job conditions and health, in order to inform possibilities for more effective social inclusion policies.

Social exclusion occurs when people lack the health, economic and cultural skills, and social support they need to gain employment and participate in the life of the community [2]. Policy based on principles of social inclusion seeks to improve people's socio-economic circumstances and their physical and mental well-being, often by encouraging them to seek employment. However, because of the complex relationships and processes involved, one should not assume that any effect of increasing workforce participation on health will be straightforward. A key to policy effectiveness is an improved understanding of the reciprocal relations between employment, work conditions and health.

### 'Stickiness' in unemployment and poor quality work

During the last decade most developed countries have experienced booming economies with low unemployment. This positive economic environment has masked a growing division between people who have prospered and those who have not [3]. Rising income inequality and growth in the number of people without living-wage jobs, point to a polarisation of labour markets into good quality employment and a pool of jobs that offer inadequate pay or conditions [3]. There have been suggestions that the rapid expansion of service employment and very low-status, low-paid jobs has reshaped the workforce into those who have good jobs and those who have bad jobs [4,5], with some adults cycling between short-term unemployment and poor quality, insecure work [3].

In addition, some people also get stuck in long-term unemployment. Duration of unemployment is a strong predictor of future employment prospects; that is, the longer someone is unemployed, the less likely they are to find work in the future [6]. Social inclusion policy should be particularly targeted at those individuals caught in cycles of disadvantage, including those who are unemployed and also those in jobs with poor working conditions.

### Poor health as a barrier to employment and good jobs

A number of personal barriers may explain the 'stickiness' associated with bad jobs and unemployment. Some barriers, such as lack of high school qualifications, poor reading skills at school, and family problems when growing up, may be selection effects that precede adverse employment circumstances [7]. Poor health is a personal characteristic that may both lead to, or follow on from employment problems. While there is strong evidence that unemployment and bad jobs are associated with poor physical and mental health, less research has examined health as a *barrier to employment*, and even less has explored health as a barrier to gaining work of reasonable quality.

There is ample cross-sectional research demonstrating that people who are unemployed generally have poorer physical and mental health than those who are employed [8]. A review by Dooley, Fielding and Levi [9] found that unemployment was associated with biomarkers of poor health such abnormal disturbances in cortisol levels [10], increased cholesterol [11] and impaired immune reactions [12], as well as unhealthy behaviours such as excessive alcohol consumption [13,14] and smoking [15]. In addition, mental health indicators such as depression [16], psychiatric admissions [17,18] and suicide [19-22] are correlated with unemployment. Research has similarly shown that those in poor quality or stressful jobs generally have worse physical and mental health than those in good quality work [23,24].

Previous research has explored whether poor health is a precursor to unemployment (i.e. whether there is a health selection effect) [1,25]. For example, chronic illness is prospectively associated with increased risk of future unemployment [26]. More recent longitudinal research [27] has found evidence of selection into unemployment based on a history of mental illness and diseases of the digestive system. While such studies indicate that poor physical and mental health may reduce the chances of selection into employment, virtually no research has examined whether health might be a barrier to gaining high quality employment. If poor physical and mental health are barriers to employment and/or good quality jobs, people could become entrenched in a cycle of disadvantage where the connection between poor health and exclusion from better quality jobs is reinforced over time. Effective social inclusion policy seeking to increase participation in community and work life needs to understand and target these compounding impacts.

### Job quality as a barrier to improved health

Although poor health may erode the chances of gaining or improving the quality of employment, the converse is also likely to be true. Having a good job could lead to health improvement - a virtuous cycle. Yet little research has examined whether gaining good quality work is likely to result in improved health. As previously mentioned, not all jobs are equal in terms of the conditions and benefits that they offer. Could it be that the positive health effects of gaining employment might depend on the quality of work obtained?

Longitudinal studies [28-30] show some support for the hypothesis that returning to work after a period of unemployment improves mental and physical health. However, stressful or adverse psychosocial work conditions have been linked to poor mental and physical health. Stressful work conditions include excessive demands, poor control, lack of job security and future job prospects, and poor workplace social support [31,32]. Research from the Whitehall II study of British civil servants has shown that physical health problems such as coronary heart disease [33,34] are linked to stress at work, and a meta-analysis [23] similarly concluded that work stressors predict psychological distress.

These findings raise the question as to whether poor psychosocial work conditions (i.e. poor quality jobs) are just as bad for mental and physical health as is unemployment. This question was investigated in a previous study that compared the mental and physical health of people at various points on a job quality continuum [24]. The results showed that mental and physical health were worse for people in jobs with greater psychosocial adversity in comparison to people in jobs with optimal work conditions. In addition, there was no significant difference in health of those who were unemployed and those in jobs with high adversity. While the work conducted by Broom and colleagues provides strong cross-sectional evidence that both unemployment and poor quality work are associated with poor physical and mental health, it is not clear whether moving from unemployment into poor quality work brings any health benefit or whether health benefits are limited to those who find good quality jobs.

### Aims and hypotheses

The current study explores how ill health might act as a barrier to gaining good quality work, and how good quality work may be an important pre-requisite for positive health outcomes. Baseline mental and physical health status were examined as potential barriers to gaining employment, and particularly gaining high quality employment. Poor job quality was examined as a barrier to improved mental and physical health amongst those who get jobs. Patterns of change in employment status and job quality across two waves of data, and the associated changes in physical and mental health were used to investigate the following three specific hypotheses:

1) There will be patterns of 'stickiness' in employment and poor quality jobs - i.e. those who are unemployed or in poor quality jobs at baseline will be more likely to remain in these circumstances at follow-up or to move between them, rather than to move into better quality jobs.

2) Poor mental and physical health status will act as a barrier to gaining employment, particularly high quality employment - i.e. those with better health at baseline will be more likely to move from unemployment into high quality jobs than those with poorer health who will be more likely to remain unemployed or move into poor quality jobs.

3) Those who move from unemployment into paid work will have improved health outcomes; however, this will only be the case for those who move into high quality jobs. In other words, job quality will determine the health effects that gaining employment bestows.

## Methods

### Participants

The PATH Through Life Project is a longitudinal, community survey assessing the health and well-being of the residents of Canberra and Queanbeyan (NSW) in Australia. It follows three cohorts of participants, initially aged 20-24, 40-44 and 60-64, interviewing them once every four years over a planned 20 year period. The current study used data from the 20s and 40s cohorts only, as work conditions were not assessed for those in the oldest cohort. Wave 1 (collected in the year 2000) participants were randomly selected from the Canberra and Queanbeyan electoral rolls. Australians aged 18 and above are required to register on the electoral roll, with few exceptions. Potential participants were drawn from a 10-year age range, as this was the minimum age range released for research purposes by the Australian Electoral Commission at this time. To contact participants aged 20-24, an introductory letter explaining the study was sent to 12414 people listed as 20-29 year olds on the electoral role. To contact participants aged 40-44 the letter was sent to 9033 people listed as 40-49 year olds. In the 20s age group, a total of 4105 people were in the correct age group and could be located, 58.6% agreed to be interviewed (n = 2404). In the 40s age group, a total of 3919 people were in the correct age group and could be located, 64.6% agreed to be interviewed (n = 2404).

At Wave 2 (collected in 2003 and 2004), 89% and 93% of participants were re-interviewed (52.1% and 60.1% of original sample) (ns = 2139, 2354). Currently, Wave 3 (collected in 2007) data are available only for the 20s cohort, where 82% of participants from Wave 1 were re-interviewed (48.2% of original sample) (n = 1978). Details for the specific sample used in the current paper are provided in the 'statistical analyses' section. Further information about the PATH sample has been previously published [35]. The PATH study was approved by the Australian National University's Human Research in Ethics Committee. Use of the data is not openly available to those outside the Chief Investigators of the project and their research teams. However, members of the research community who are interested in using the data can apply to the PATH governance committee for access.

**Figure 1:**
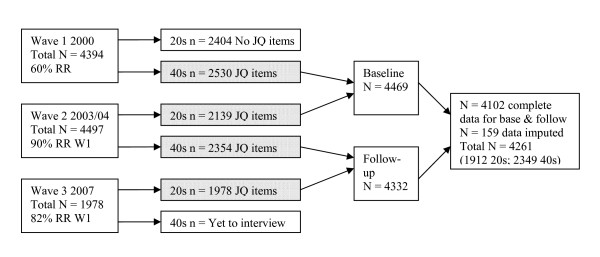
**Participation rates and sample groups across the three waves of data (JQ = job quality; RR = response rate)**.

Each of the 20s and 40s cohorts has two time points of data about work conditions available (which we labelled 'baseline' and 'follow-up' for this analysis). For the 40s cohort, data is available on work conditions at Waves 1 and 2 of data collection (2000 and 2004), while for the 20s cohort questions about work conditions were included at Waves 2 and 3 (2003 and 2007). Comparable data from both the 20s and 40s cohorts were pooled in the current study to maximise the power to detect effects. So, due to differences in when the job quality data was collected, 'baseline' and 'follow-up' represent data from Waves 2 and 3 for the 20s cohort, and Waves 1 and 2 for the 40s cohort. Figure 1 provides a flow chart of the number of participants in the 20s and 40s sample groups across the waves, and how data from each wave and age group were combined.

### Survey Procedure

For Wave 1, persons were randomly selected from the electoral roll and sent a letter with information about the survey, explaining that an interviewer would contact them. A convenient time and place for the interview was arranged for those who agreed to participate. The interview took one-and-a-half to two hours and was usually conducted at the person's home or at the Centre for Mental Health Research in Canberra. The interviewer took the participant through the first set of questions, demonstrating how to enter responses into a palmtop personal computer using Surveycraft software. The majority of the survey was then completed by the respondent alone, including the work conditions questions. For Wave 2 of the survey, participants were re-contacted by telephone approximately 4 years later and asked whether they would participate in the second wave of the study. This procedure was repeated for Wave 3. A consistent interview process was used across all waves.

### Measures

The measures used in this paper and described below were consistent across all time points of data collection. Measures were dichotomised in a number of cases to replicate those used in previous, related published works [24,31].

### Employment and job quality measures

*Employment status *consisted of three categories to represent current labour force participation. Participants were coded as '1' if they were employed either full or part-time, as '2' if they were unemployed and looking for work, and '3' if they were not participating in the labour force.

A job adversity measure was created by combining three key measures of work related psychosocial stress; job strain, job insecurity and job marketability (each of these measures is described below).

*Job strain *is a measure of both job demands and job control. 4 items assessed job demands, such as "Do you have to work very fast?", and 15 items assessed job control, such as "Others take decisions concerning my work". There were four response categories available for each item, 1 "often", 2 "sometimes", 3 "rarely" and 4 "never". These 19 items were taken from the Whitehall study [36], which originally adapted the items from the Job Content Questionnaire [37]. Total scale scores for job demands and job control were created by averaging the total scores of the relevant items. Following the methodology adopted by Broom, D'Souza, Strazdins et al. [24] and Strazdins, D'Souza, Lim, et al. [31], these scales were then dichotomised at the median (20s cut-offs for both time points: demands = 3.11, control = 2.75; 40s cut-offs for both time points: demands = 3.22, control = 2.75), and the two variables were combined to create a measure of job strain. High job strain "1" was defined as low job control (below the median scale score) and high job demands (above the median scale score), whereas all other combinations indicated low job strain "0".

Perceived *job insecurity *was measured by the question "How secure do you feel about your job or career future in your current workplace?" Possible responses were 1 "not at all secure", 2 "moderately secure", 3 "secure" and 4 "extremely secure". Those who answered either "not at all" or "moderately secure" were categorised as having high job insecurity "1", while the other categories represented low job insecurity "0". Perceived *job marketability *(ability to get another job) was measured using the question "If you lost your present job, how difficult do you think it would be to get another job with the same pay and hours?" Response options were: 1 "not at all difficult", 2 "moderately difficult", 3 "difficult", 4 "extremely difficult". Those who said finding another job would be difficult or extremely difficult were categorised with low marketability "1". As in Boom, D'Souza, Strazdins et al. [24], we included measures of job insecurity and job marketability in our overall measure of job quality, because of their relevance to (and ability to somewhat account for) the influence of the contemporary labour market.

An overall measure of *job quality *or an *employment continuum *was created by summing the reported number of adverse work conditions (high job strain, high job insecurity and low job marketability) and ranged from 0 to 3. Further details on this measure of job quality are available in Broom, D'Souza, Strazdins et al. [24] and see Grzywacz and Dooley [38] for a similar approach. Previous research conducted by Strazdins, D'Souza, Lim, et al. [31] supports the approach of constructing a composite measure. This research suggests that pooled measures of job strain and insecurity provide a better description of contemporary work stress than either measure alone. In all analyses except the replication of Broom et al. (Study 2, Table 1), this employment continuum was recoded into two categories to ensure adequate cell sizes: a) those participants who experienced either 0 or 1 adverse condition ("0" low job adversity), and b) those participants who experienced either 2 or 3 adverse conditions ("1" high job adversity). It should be noted that although the term job quality is used throughout this paper to refer to the composite measure of job characteristics, the marketability item is also closely related to individual qualifications and labour market conditions.

**Table 1:** Employment pathways in association with health status at follow-up (n = 90).

	Follow-up health status		
	**Depression OR (95% CI)**	**Anxiety OR (95% CI)**	**Physical Health OR (95% CI)**

Employment pathway from baseline to follow-up^a^			

Pathway 1 (high quality)	1.00	1.00	1.00

Pathway 2 (low quality)	5.43 (2.17-13.58)**	1.91 (.80-4.56)	.68 (.22-2.13)

Pathway 3 (unemployed)	1.58 (.57-4.40)	2.08 (.73-5.94)	1.70.53-5.46)

Notes. Calculated using Logistic Regression. Imputed data used for calculations. * Significance <.05, ** Significance <.001. The model shown was adjusted for baseline health status, gender, age group, education and partner status.

### Health measures

*Depression *and *anxiety *were assessed using the Goldberg Anxiety and Depression Scales [39]. Each scale consists of nine items that assess psychiatric symptoms. These were summed to give two total scale scores ranging from 0 to 9. As in Broom, D'Souza, Strazdins et al. [24] cut-offs close to the upper octile were used to dichotomise the continuous scores for each scale. The nearest cut-off for the depression subscale (>6 for both age groups) identified 15% of respondents as depressed at baseline, and 14% as depressed at follow-up. The nearest cut-off for the anxiety subscale (>7 for both age groups) identified 18% of respondents as anxious at baseline, and 17% as anxious at follow-up. These prevalence rates are reasonable indicators of significant symptomology and approximate clinical diagnostic criteria. The 2007 Australian National Survey of Mental Health found that the 12 month prevalence of any affective disorder was 6.2% and the 12 month prevalence of any anxiety disorder was 14.4% [40]. These rates increase to 8.0% and 17.3% when restricted to an age range of 24-48.

The short-form physical health summary (SF-12) was used to assess *physical functioning *[41]. Similar to the mental health measures, scores for the SF-12 were dichotomised close to the lower octile (<43 for both age groups), identifying 11% as having physical health problems at baseline and 13% at follow-up.

### Covariates

Demographic measures adjusted for in the analyses (at baseline) were *gender*, *years of education *and *marital/partner status *(1 "married/de facto", 2 "separated/divorced/widowed", and 3 "never married"). *Major life events *in the last 6 months, including serious injury/illness, death of a close family member, and relationship problems (1 "experienced" and 0 "not experienced"), were also controlled for in the analyses. In the absence of information about income, participants were asked about their *financial circumstances*: "Have you or your family had to go without things you really needed in the last year because you were short of money?" Possible response options were: 1 "yes often", 2 "yes sometimes" and 3 "no". As *negative affectivity *has been previously linked to work stress and self-reported health this personality trait was assessed and adjusted for using the 7 item Behavioural Inhibition Scale (BIS) [42].

### Statistical analyses

A large majority of participants (n = 4102, 88%) had complete data at both baseline and follow-up. 398 participants (9%) dropped out of the survey after baseline, and were excluded from the analyses. 56% of this group of non-respondents were in the 20s age group, 54% were male, 5.5% were unemployed at time 1, and their average scores on the Goldberg Depression and Anxiety scales were 2.96 and 3.86. To minimise concern about attrition leading to biased findings, data from these participants were used to weight the analyses to account for their exclusion. Inverse probability weights were calculated by using variables relevant to the analyses (time 1 employment, job quality, mental health, life events, marital status, financial problems) to predict participant drop-out after baseline.

A further 10 cases were omitted due to missing data on more than 25% of the variables included in the analyses. The full set of variables was used to impute missing data for a further 159 cases, with 80% of these cases requiring imputation of two or fewer variables. Missing data were imputed using the expectation-maximisation algorithm is SPSS MVA procedure in version 17.0. The algorithm used is described by Enders [43]. The key assumption in this approach is the missingness is either completely random or can be predicted from observed values (missing at random; MAR). MAR is an untesTable 1ssumption but given the small proportion of data imputed, the effect of any violation of the MAR assumption would be negligible. The final sample included was 4261 (1912 from the 20s age group and 2349 from the 40s age group) with 47% male and 53% female (see Figure 1). The hypotheses under investigation were tested in three sub-studies:

### Study 1: stickiness of unemployment and low quality jobs

Initially, cross-tabulations examined the number of participants within each of the employment and job condition categories at baseline and follow-up, and the transitions across time points. This analysis compared the number of participants found to make each transition, with the number of participants expected to make the transition based on chance (expected cell frequencies). This first study included 'not in the labour force' as an employment category to provide descriptive information about the number of people moving in and out of this state. However, as 'not in the labour force' is a heterogeneous group in terms of life circumstances and health status, respondents in this category at either baseline or follow-up were not included in studies 2 and 3. A separate, detailed investigation should be undertaken at a later stage to explore the barriers specific to moving into and out-of the labour force, and the effects on health, taking account of the heterogeneity of this group.

### Study 2: health barriers to gaining employment

Multinomial logistic regression (MLR) was used to examine physical and mental health as barriers to gaining employment, particularly high quality employment, for those initially unemployed; that is whether baseline mental and physical health status predicted the type of employment pathways between baseline and follow-up. Those who moved from unemployment into high quality jobs were compared to those who remained unemployed and those who moved into poor quality jobs. All MLRs in Study 2 (Table 2) adjusted for basic socio-demographic factors (gender, age group, education, partner status). Other potential confounders (financial problems, negative affectivity, serious injury/illness/assault death of a close family relative and relationship problems) were not included as the limited sample size introduced to a lack of variability (singularity) in these factors. In addition, supplementary univariate analyses suggested their contribution to the MLR models was minimal.

**Table 2:** Baseline health status in association with pathways from unemployment (n = 90).

	Employment pathway from unemployment at baseline		
	**Pathway 1 To high quality jobs OR (95% CI)**	**Pathway 2 To low quality jobs OR (95% CI)**	**Pathway 3 Remaining unemployed OR (95% CI)**

Baseline health status			
Depression	1.00	7.39 (3.16-17.27)**	2.56 (.94-6.99)
Anxiety	1.00	1.87 (.78-4.50)	2.93 (1.08-7.96)*
Poor physical health	1.00	.76 (.19-2.9)	5.83 (1.72-19.81)*

Notes. Calculated using Multi-nomial Logistic Regression. Imputed data used for calculations. * Significance <.05, ** Significance <.001. The model shown was adjusted for baseline health status, gender, age group, education and partner status.

### Study 3: health outcomes following movement into paid work

Logistic regression (LR) models examined the effect of employment status on health status at follow-up, with adjustments for the effects of employment status and health at baseline. LR was also used to examine the effect of job quality on health status at follow-up, again with adjustments made for baseline status. This second analysis compared the highest job quality category (employed with no adverse work conditions) with the other three categories of job quality (1-3 adverse work conditions) and with the unemployed group, and a further comparison was made between the unemployed and the '3 adverse work conditions' category. These models (shown in Tables 3 and 4) adjusted for all potential confounders (gender, age group, education, partner status, financial problems, negative affectivity, serious injury/illness/assault death of a close family relative and relationship problems).

**Table 3:** Employment status at follow-up in association with health status at follow-up (n = 3755).

Employment continuum at T2	Depression OR (95% CI)	Anxiety OR (95% CI)	Poor physical health OR (95% CI)
Employed	1.00	1.00	1.00
Unemployed	2.33 (1.35-4.03)*	1.41 (.80-2.47)	2.33 (1.38-3.94)*

Notes. Calculated using Logistic Regression. Imputed data used for calculations. * Significance <.05, ** Significance <.001. The model shown was adjusted for baseline employment and health status, age group, gender, education, marital status, financial problems, negative affectivity (behavioural inhibition), serious injury/illness/assault, death of a close family relative and relationship problems.

**Table 4:** Employment continuum at follow-up in association with health status at follow-up (n = 3755).

Employment continuum at T2	Depression OR (95% CI)	Anxiety OR (95% CI)	Poor physical health OR (95% CI)
0 adverse conditions	1.00	1.00	1.00
1 adverse conditions	2.03 (1.56-2.63)**	1.64 (1.30-2.07)**	1.57 (1.22-2.02)**
2 adverse conditions	2.58 (1.87-3.56)**	2.45 (1.83-3.29)**	1.25 (.89-1.76)
3 adverse conditions	5.24 (3.08-9.57)**	3.23 (1.87-5.59)**	1.91 (1.06-3.42)*
Unemployed	4.12 (2.34-7.27)**	2.16 (1.21-3.86)**	2.95 (1.71-5.09)**

Notes. Calculated using Logistic Regression. Imputed data used for calculations. * Significance <.05, ** Significance <.001. The model shown was adjusted for baseline employment and health status age group, gender, education, marital status, financial problems, negative affectivity (behavioural inhibition), serious injury/illness/assault, death of a close family relative and relationship problems.

Further analyses examined whether change in employment status across two waves predicted change in physical and mental health status. These analyses focused on identifying the physical and mental health consequences of pathways from unemployment. LR was used to examine whether mental and physical health at follow-up was influenced by the type of employment transition undertaken. This final set of LRs (Table 1) adjusted for basic socio-demographic factors (gender, age group, education, partner status).

## Results

### Descriptive statistics

Table 5 provides descriptive statistics on employment status and job quality within the sample. At both baseline and follow-up the majority of respondents were employed. Only 2.6% of participants at baseline and 2.0% at follow-up were unemployed, and 7.3% and 8.0% were not in the labour force at each time point. Relatively few participants experienced 2 adverse work conditions (17.1% at baseline; 15.1% at follow-up), and still fewer experienced 3 (3.8% at baseline; 2.9% at follow up). Most experienced 0 or 1 adverse job conditions.

**Table 5:** Employment and job quality status at baseline and follow-up.

	Baseline (n = 4261)	Follow-up (n = 4261)
	
	Employed = 3843 (90.2%)	Employed = 3836 (90.0%)
Employment	Unemployed = 109 (2.6%)	Unemployed = 84 (2.0%)
	Not in LF = 309 (7.3%)	Not in LF = 341 (8.0%)
	
	Baseline (n = 3843*)	Follow-up (n = 3836*)
	
	0 adverse cond. = 1636 (42.6%)	0 adverse cond. = 1728 (45.0%)
Continuum	1 adverse cond. = 1404 (36.5%)	1 adverse cond. = 1418 (37.0%)
(median split)	2 adverse cond. = 656 (17.1%)	2 adverse cond. = 573 (15.1%)
	3 adverse cond. = 147 (3.8%)	3 adverse cond. = 111 (2.9%)

Notes. Imputed data used for calculations. * Subgroup of respondents who were employed. Adverse condition is a count of high job strain, high job insecurity and low marketability.

### Study 1: stickiness of unemployment and low quality jobs

Table 6 shows that the majority of participants remained stable in their employment situations, with 93.9% of those who were employed at baseline remaining employed at follow-up, 13.8% of those unemployed remaining so, and 46.6% of those not in the labour force remaining so (in all three cases this was significantly more than the expected frequencies for these cells). The majority of people not in the labour force across both time points were female (86.8%) and in the 40s age group (67.4%). Fewer people than expected moved from employment to unemployment (observed n = 57 vs. expected n = 76) or employment to not in the labour force (observed n = 178 vs. expected n = 308); however more people than expected moved between the categories of unemployment and not in the labour force (observed n = 19 vs. expected n = 9; observed n = 12 vs. expected n = 6).

**Table 6:** Employment status and job condition transitions from baseline to follow-up (n = 4261).

	Follow-up					
	**Employed**	**High qual. jobs**^**a**^	**Low qual. jobs**^**b**^	**Unemployed**	**Not in LF**	**Total**

Baseline						

Employed	**3608 (93.9%)** **(Exp: 3460)**	-	-	**57 (1.5%)** **(Exp: 76)**	**178 (4.6%)** **Exp: 308)**	**100%**

High quality jobs^a^	-	**2537 (83.5%)** **(Exp: 2278)**	**324 (10.7%)** **(Exp: 459)**	**42 (1.4%)** **(Exp: 60)**	**137 (4.5%)** **(Exp: 243)**	**100%**

Low quality jobs^b^	-	**477 (59.4%)** **(Exp: 602)**	**270 (33.6%)** **(Exp: 121)**	15 (1.9%) (Exp: 16)	**41 (5.1%)** **(Exp: 64)**	**100%**

Unemployed	**75 (68.8%)** **(Exp: 98)**	**54 (49.5%)** **(Exp: 82)**	21 (19.3%) ((Exp: 16)	**15 (13.8%)** **((Exp: 2**)	**19 (17.4%)** **((Exp: 9)**	**100%**

Not in Labour Force	**153 (49.5%)** **((Exp: 278)**	**125 (40.5%)** **((Exp: 232)**	**28 (9.1%)** **(Exp: 47)**	**12 (3.9%)** **(Exp: 6)**	**144 (46.6%)** **(Exp: 25)**	**100%**

Notes. Calculated using cross-tabulations. Imputed data used for calculations. Exp: Expected count of cell frequencies. Bold figures indicate significant differences between observed and expected cell values (p < .05). This was determined with an adjusted residual score <-2.00 or >2.00 (Agresti, 1996 - [53]). a) 0 or 1 adverse conditions, b) 2 or 3 adverse conditions.

Table 6 also shows the number of people who made each employment status and job quality transition from baseline to follow-up. The second and third rows are of primary interest. The third row reports the transitions made by those initially employed in low quality jobs (2 or 3 adverse conditions). The data show that fewer of these people moved into high quality jobs (with 0 or 1 adverse condition) than would be expected by chance alone (observed n = 477 and expected n = 602). Also, twice as many people as expected remained in low quality jobs across both time points (observed n = 270 and expected n = 121). The fourth row reports the transitions made by those initially unemployed. Similarly, the data indicate that fewer people moved from unemployment into high quality jobs than would be expected by chance alone (observed n = 54 and expected n = 82) and more people than expected remained unemployed across both time points (observed n = 15 and expected n = 2). It was also hypothesised that there would be a tendency for people to move between states of unemployment and low quality jobs, but this was not found to be the case (see non-bolded cells).

### Study 2: health barriers to gaining employment

This study reports analyses which investigate whether the type of employment transition from baseline to follow-up was predicted by baseline health status.

**Figure 2:**
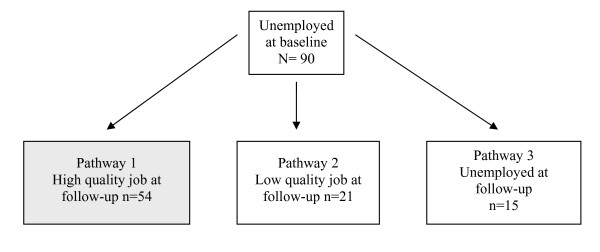
Numbers of participants in each pathway from baseline to follow-up

Table 2 assesses whether the physical and mental health status of those unemployed at baseline is associated with subsequent employment status, examining the three pathways shown in Figure 2. In comparison to those who moved into a high quality job, those who took a low quality job were more likely to be depressed (*OR *= 7.39) at baseline. Those who remained unemployed were also more likely to be anxious (*OR *= 2.93) and have physical health problems (*OR *= 5.83).

A further analysis directly compared those who remained unemployed and those who transitioned into poor quality jobs. The findings showed that, compared to those who remained unemployed, those who transitioned into poor quality jobs were more likely to be depressed at baseline (*OR *= 2.89 (95% *CI *= 1.01-8.25)), but less likely to have physical health problems (*OR*=.13 (95% *CI*=.03-.60)).

### Study 3: health outcomes following movement into paid work

Next we extend the analyses first reported by Broom, D'Souza, Strazdins et al. (2006), considering health status by employment status and job quality. In comparison, our analyses controlled for baseline health (a potential major confounder) and added in a second age cohort. The data shown in Table 3 compares the health of those who were employed with those who were unemployed, adjusting for baseline employment and health status, and additional potential confounders. The odds of experiencing depression were significantly greater among those who were unemployed than for those who were employed (*OR *= 2.33). The results for anxiety were not significant (*OR *= 1.41). The odds of having poor physical health were also significantly greater for the unemployed (*OR *= 2.33) than the employed. No interactions were found between employment status and age group, or between employment status and gender, for any of the health variables.

Table 4 compares the health status at follow-up of people in jobs with no adverse conditions with people experiencing one, two or three adverse psychosocial job conditions and those who were unemployed, again adjusting for baseline work and health characteristics. Experiencing psychosocial job adversities and being unemployed were associated with poorer physical and mental health status than experiencing no work adversity. For both depression and anxiety, the odds of poor health were greatest for those experiencing three adverse working conditions. For physical health, the odds of experiencing poor health were greatest for the unemployed. Again, no interactions were found between employment status and age group, nor employment status and gender for any outcome. In testing for differences between those who had the poorest quality jobs (3 adverse conditions) and the unemployed, no significant differences were shown for depression, anxiety or physical health.

**Figure 3:**
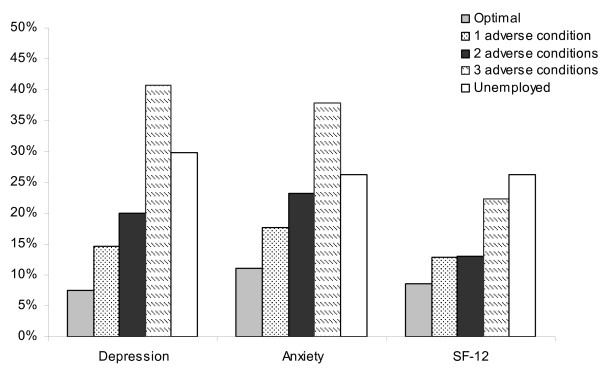
Percentage of participants characterised as having a health problem in each employment group

The findings from Table 4 are supported by Figure 3. The figure indicates that the percentage of participants with mental and physical health problems at follow-up increased with the number of job adversities experienced and that those who were unemployed reported similar levels of physical and mental health problems as those in the poorest quality jobs.

Further analyses investigated whether the type of transition in employment status (moving from unemployment into a high quality job versus alternative transitions) predicted follow-up health status.

In comparison to respondents who moved from unemployment into high quality jobs (pathway 1), those who moved into low quality jobs were more likely to report experiencing depression at follow-up (*OR *= 5.43). A direct comparison was also made between those who remained unemployed and those who moved into poor quality jobs. The findings(Table 1) showed that those who took up poor quality jobs were significantly more likely to be depressed at follow-up than those who remained unemployed (*OR *= 3.44 (95% *CI=*1.14-10.39)).

## Discussion

The current study provides insight into the complexities surrounding the relationships between employment, physical and mental health, and job quality. Each of the hypotheses proposed received some support. First, we did find some evidence of patterns of 'stickiness' in employment and poor quality jobs. Those respondents who were unemployed at baseline were at increased risk to be unemployed at follow-up and, similarly, those in poor quality jobs at baseline were also likely to be in poor quality work at follow-up. However, contrary to expectations, there was no indication that being unemployed was associated with increased risk of being in a poor quality job. Second, baseline mental and physical health status predicted employment transitions - indicating they may act as barriers to gaining high quality employment. Specifically, in comparison to those who moved into high quality work, those who remained unemployed were more likely to have symptoms of anxiety and physical health problems at baseline, and those who moved into low quality work were more likely to be depressed at baseline. Further, those who transitioned into poor quality work from being unemployed were more likely to be depressed at baseline (than those who remained unemployed). Third, it was indicated that the quality of work people enter into influences their subsequent mental health status. Those who moved from unemployment into poor quality work were more likely to be depressed at follow-up than those who moved into high quality work.

In combination, these findings shed light on the important and complex inter-relationships between work and health. First they confirm that the relationships between work, health and job quality are not straightforward. While this is not a new discovery, it is often dealt with poorly or ignored in studies of employment. Dooley and Catalano [44] previously noted that a "comprehensive theory for the unemployment-health relationship would include multiple direct, indirect, reciprocal, and interacting causal pathways" [[2], p.g. [30]]. We found that not only does health status influence the type of employment transition people undertake but that at the same time, the type of transition undertaken influences health status. While these results may seem to achieve little in terms of defining causality, they accurately reflect the cyclic nature of the associations involved, and the interplay that occurs between exposure and selection effects [1,45]. Tellingly, previous research has similarly shown that health affects employment opportunities (health selection) [26,27], that employment conditions affect health (exposure) [28,29], and that job quality is associated with health [24]. The current study is one of the first to show all three of these findings in the one sample group.

Although the relationships between work, health and job quality are dynamic and reciprocal in nature, the current paper illustrates there is some useful predictability in terms of who goes where, and why. Study 1 examined 'stickiness' in patterns of employment and did show that there was consistency in adverse employment circumstances over time. Those who were initially unemployed were more likely to unemployed; those who were initially in poor quality jobs were at increased risk of being subsequently identified in poor quality jobs. This reflects suggestions made in Dooley and Prause [3] that it is becoming more difficult or uncommon for people to move out of their 'employment class' whether it be, unemployment, poor quality work or high quality work. However, our hypothesis that labour market disadvantage would be evident in the clustering of employment in poor quality jobs and unemployment within individuals was not supported. Thus, the current study provides no evidence that there are certain individuals who cycle between unemployment and poor quality jobs. Study 2 provides one explanation for stickiness by demonstrating that baseline health status predicts the type of future employment transitions undertaken. It suggests that if people are unemployed and experiencing symptoms of anxiety or poor physical health they are more likely to remain unemployed than to move into good quality work. Transitioning from unemployment into poor quality work was also found to be strongly associated with baseline depression. Study 3 highlights the health benefits of good quality work. The findings showed that in comparison to those who gained good quality work after unemployment, the odds of having depression at follow-up were 5 times greater for those who moved to poor quality jobs.

Differences in the findings for depression and physical health status are worth noting. Both studies 2 and 3, found that depression was strongly associated with poor quality work, whether baseline depression predicted transition into poor quality work (study 2) or whether the transition into poor quality work predicted depression (study 3). Although these findings are related, they do seem to reflect two separate effects as the differences in subsequent depression remained after controlling for baseline depression. Study 2 also showed that baseline physical health status was closely associated with persistent unemployment. Together these findings hint that it may be poor physical health status that best defines long-term unemployment, and depression that characterises the people trapped in bad jobs or cycling between bad jobs and unemployment. However, future research with more extended longitudinal data and more participants in each sub-cohort group would be necessary to confirm this hypothesis.

### Practical implications

The current study has implications for the development of workforce policy and raises new questions for future research. A common component of governments' social inclusion, participation and economic agendas is finding work for the unemployed. The Australian Government report *Social Inclusion: Origins, Concepts and Key Themes *names 'securing a job' as the key component of social inclusion [46]. The current results suggest that interventions and re-employment programs designed to decrease unemployment should be aware that health is associated with people's likelihood of being caught in patterns of unemployment or poor quality work. Poor health status appears to reduce people's ability to access and retain a good quality job. However, if they succeed in obtaining a good quality job, their health may improve (a key goal of social inclusion policy). Policy makers should recognise that health and social characteristics may affect the likely success and quality of employment outcomes achieved. More broadly, workplace policies which focus on preventing and/or decreasing employment stressors, such as job strain, or on facilitating transition to better quality jobs are more likely to benefit employee's physical and mental health status, and reduce the burden of illness on public health systems.

### Limitations and strengths

Several potential limitations should be considered. First, although the current sample was representative of the population from which it was recruited (Canberra and Queanbeyan, Australia), this region is not necessarily representative of the broader Australian population. Canberra residents have been shown to have higher average weekly incomes and labour force participation and employment rates than the national average [47,48]. ABS 2006 census data shows that the profile of the workforce in Canberra/Queanbeyan has almost 50% more professionals and much lower rates of persons employed in sales, machinery operators/driver & labourers than the overall Australian population [49]. The PATH sample contained few people who were unemployed or in poor quality jobs and the cohort groups used to investigate the effect of baseline characteristics on employment transitions were small. As a result, the power to detect significant effects within the current study was limited in comparison to a study that may contain a more economically and occupationally diverse sample group. However, it is encouraging to note that the rates of unemployment in the PATH sample are comparable to Canberra/Queanbeyan unemployment rates from the Australian Bureau of Statistics at the time the surveys were conducted (20s baseline 6.89%; 20s follow-up 2.46%; 40s baseline 2.72%; 45s follow-up 1.73%) [49]. Although these figures suggest there is not an under-representation of unemployed people in the PATH data when compared with the local community, given the non-response rate for the survey (approx. 60% - comparable with other response-rates in published medical journals [50]) the risk of bias in the sample and in attrition (particularly regarding socio-economic factors [51]) cannot be discounted.

A second limitation is that only two time points, spaced four years apart without any information between these time points, were available for analysis in the current study. As such, the 'baseline' point in the data does not necessarily reflect the commencement of either unemployment or health problems, making it difficult to tease out the direction of causal relationships. Shorter time periods between waves with additional time points would provide a more informative picture of the types of employment transitions people make, and their potential impact on health. Additional time points would also provide detail on duration of unemployment, an important factor that could not be assessed in the current study.

A further limitation is that the measures of physical and mental health and poor job quality were self-report. As such, response-bias or endogeneity may play a role in the significant associations found between health and job quality [52]. This possibility was minimised in the analyses by including negative-affect as a potential covariate. In addition, although the cut-off points used from the self-report measures replicated those adopted in previous published work, and were epidemiologically credible and theoretically reasonable, they are not equivalent to objective measures of job quality or diagnostic measures of mental health. Finally, men and women were combined in the current study to maximise the power to detect significant effects. It is likely that men and women have different reasons for spells of unemployment or poor quality work. Gender interactions are tested throughout the paper to rule out substantial gender differences; however this is an area where further research is required.

Despite these limitations, there are a number of strengths to the current study. Longitudinal studies rarely contain valid measures of employment status and job quality, as well as a range of potential covariates. The unique combination of measures in this study allowed for the adjustment of several covariates known to affect employment status and health outcomes, including negative affectivity and years of education, strengthening the validity of the findings. The adjustment for education is particularly important, as it is possible that links between unemployment and low quality work are centrally due to low levels of education. The significant associations shown in the current study, post-adjustment, suggest there is a strong independent association between mental health and employment circumstances. In addition, the two time points of data available allowed the research to move beyond cross-sectional associations, and investigate the relationship between health and employment transitions.

## Conclusions

Our research shows that the relationships and processes involved in increasing workforce participation and improving health are complex and inter-related. However, it is necessary to tease out the important elements involved in these processes in order to inform effective policies for employment and social exclusion. The current findings show that people are more likely to remain in their current employment circumstances, including unemployment and poor quality work, than to move out of them, and that this association is at least partly predicted by mental and physical health status. The likely best case scenario for people who are unemployed is to gain a good quality job - i.e. a job that is secure, provides future job prospects, and has low levels of strain. Doing so increases their chances of good mental health, and decreases their chances of becoming entrenched in the cycle of unemployment, adverse jobs and poor health status.

## Competing interests

The authors declare that they have no competing interests.

## Authors' contributions

All authors participated in the original conception and design of the study. LL conducted the statistical analyses. PB and BR were involved in the design and acquisition of the survey data. LL, PB and LS wrote the initial drafts of the paper. PB, LS, DB, BR and SO provided comments on the manuscript. All authors read and approved the final manuscript.

## Pre-publication history

The pre-publication history for this paper can be accessed here:

http://www.biomedcentral.com/1471-2458/10/621/prepub

## References

[B1] Bartley M Unemployment and health: selection or causation - a false antithesis? Sociol Health Illn 1988 10 41 67 10.1111/1467-9566.ep11340114

[B2] Atkinson T Cantillon B Marlier E Nolan B Social Indicators: The EU and Social Inclusion 2002 United Kingdom, Oxford, Oxford University Press

[B3] Dooley D Prause J The Social Costs of Underemployment 2004 United Kingdom, Cambridge University Press

[B4] Lindsay C McQuaid RW Avoiding the 'McJobs': Unemployed job seekers and attitudes to service work Work, Employ Soc 2004 18 297 318 10.1177/09500172004042771

[B5] Sassen S Mingione E Service employment refimes and the new poverty Urban Poverty and the Underclass 1996 Oxford, Blackwell

[B6] Jackman R Laylard R Does Long-Term Unemployment Reduce a Person's Chance of a Job? A Time-Series Test Economica 1991 58 229 93 106 10.2307/2554977

[B7] Caspi A Wright BR Moffitt TE Silva PA Early failure in the labor market: Childhood and adolescent predictors of unemployment in the transition to adulthood Am Sociol Rev 1998 63 424 451 10.2307/2657557

[B8] Jin RL Shah CP Svoboda TJ The impact of unemployment on health: a review of the evidence Can Med Assoc J 1995 153 5 529 540 PMC1487417 7641151

[B9] Dooley D Fielding J Levi L Health and unemployment Ann Rev Public Health 1996 17 449 465 10.1146/annurev.pu.17.050196.002313 8724235

[B10] Brenner MH Economic instability, unemployment rates, behavioral risks, and mortality rates in Scotland, 1952-1983 Int J Health Services 1987 17 3 475 487 10.2190/5GVU-86Y6-NH1U-PQB0 3623777

[B11] Levi L Work, stress and health Scand J Work Environ Health 1984 10 6 495 500 6535251 10.5271/sjweh.2295

[B12] Arnetz BB Brenner SO Levi L Hjelm R Petterson IL Wasserman J Petrini B Eneroth P Kallner A Kvetnansky R Neuroendocrine and immunologic effects of unemployment and job insecurity Psychother Psychosom 1991 55 2-4 76 80 10.1159/000288412 1891571

[B13] Dooley D Catalano R Hough R Unemployment and alcohol disorder in 1910 and 1990: drift versus social causation J Occup Organ Psychol 1992 65 277 290

[B14] Janlert U Hammarstrom A Alcohol consumption among unemployed youths: results from a prospective study Br J Addict 1992 87 5 703 714 10.1111/j.1360-0443.1992.tb02716.x 1591521

[B15] Lee A Crombie IK Smith WC Tunstall-Pedoe HD Cigarette smoking and employment status Soc Sci Med 1991 33 11 1309 1312 10.1016/0277-9536(91)90080-V 1776044

[B16] Dooley D Catalano R Wilson G Depression and unemployment: panel findings from the Epidemiologic Catchment Area study Am J Community Psychol 1994 22 6 745 765 10.1007/BF02521557 7639201

[B17] Lajer M Unemployment and hospitalization among bricklayers Scand J Soc Med 1982 10 1 3 10 7112053 10.1177/140349488201000102

[B18] Kiernan M Toro PA Rappaport J Seidman E Economic predictors of mental health service utilization: a time-series analysis Am J Community Psychol 1989 17 6 801 820 10.1007/BF00922739 2636540

[B19] Platt S Unemployment and suicidal behaviour: a review of the literature Soc Sci Med 1984 19 2 93 115 10.1016/0277-9536(84)90276-4 6382623

[B20] Morrell S Taylor R Quine S Kerr C Suicide and unemployment in Australia 1907-1990 Soc Sci Med 1993 36 6 749 756 10.1016/0277-9536(93)90035-3 8480219

[B21] Pritchard C Is there a link between suicide in young men and unemployment? A comparison of the UK with other European Community Countries Br J Psychiatry 1992 160 750 756 10.1192/bjp.160.6.750 1617355

[B22] Fairweather AK Anstey KJ Rogers B Butterworth P Factors distinguishing suicide attempters from suicide ideators in a community sample: social issues and physical health problems Psychol Med 2006 36 9 1235 1245 10.1017/S0033291706007823 16734946

[B23] Stansfeld S Candy B Psychosocial work environment and mental health--a meta-analytic review Scand J Work Environ Health 2006 32 6 443 462 17173201 10.5271/sjweh.1050

[B24] Broom DH D'Souza RM Strazdins L Butterworth P Parslow R Rodgers B The lesser evil: bad jobs or unemployment? A survey of mid-aged Australians Soc Sci Med 2006 63 3 575 586 10.1016/j.socscimed.2006.02.003 16597477

[B25] Mastekassa A Unemployment and health: Selection effects J Appl Soc Psychol 1996 6 189 205 10.1002/(SICI)1099-1298(199608)6:3<189::AID-CASP366>3.0.CO;2-O

[B26] Arrow JO Estimating the influence of health as a risk factor on unemployment: a survival analysis of employment durations for workers surveyed in the German Socio-Economic Panel (1984-1990) Soc Sci Med 1996 42 12 1651 1659 10.1016/0277-9536(95)00329-0 8783427

[B27] Heponiemi T Elovainio M Manderbacka K Aalto AM Kivimaki M Keskimaki I Relationship between unemployment and health among health care professionals: health selection or health effect? J Psychosom Res 2007 63 4 425 431 10.1016/j.jpsychores.2007.04.005 17905052

[B28] Reine I Novo M Hammarstrom A Does transition from an unstable labour market position to permanent employment protect mental health? Results from a 14-year follow-up of school-leavers BMC Public Health 2008 8 159 10.1186/1471-2458-8-159 18477384 PMC2409329

[B29] Strandh M Different exit routes from unemployment and their impact on mental well-being: the role of the economic situation and the predictability of the life course Work Employ Soc 2000 14 3 459 479

[B30] Vuori J Silvonen J The benefits of a preventive job search program on re-employment and mental health at 2-year follow-up J Occup Organ Psychol 2005 78 43 52 10.1348/096317904X23790

[B31] Strazdins L D'Souza RM Lim LL Broom DH Rodgers B Job strain, job insecurity, and health: rethinking the relationship J Occup Health Psychol 2004 9 4 296 305 10.1037/1076-8998.9.4.296 15506847

[B32] Griffin JM Greiner BA Stansfeld SA Marmot M The effect of self-reported and observed job conditions on depression and anxiety symptoms: a comparison of theoretical models J Occup Health Psychol 2007 12 4 334 349 10.1037/1076-8998.12.4.334 17953493

[B33] Chandola T Britton A Brunner E Hemingway H Malik M Kumari M Badrick E Kivimaki M Marmot M Work stress and coronary heart disease: what are the mechanisms? Eur Heart J 2008 29 5 640 648 10.1093/eurheartj/ehm584 18216031

[B34] Chandola T Brunner E Marmot M Chronic stress at work and the metabolic syndrome: prospective study Br Med J 2006 332 7540 521 525 10.1136/bmj.38693.435301.80 PMC1388129 16428252

[B35] Jorm AF Butterworth P Anstey KJ Christensen H Easteal S Maller J Mather KA Turakulov RI Wen W Sachdev P Memory complaints in a community sample aged 60-64 years: associations with cognitive functioning, psychiatric symptoms, medical conditions, APOE genotype, hippocampus and amygdala volumes, and white-matter hyperintensities Psychol Med 2004 34 8 1495 1506 10.1017/S0033291704003162 15724880

[B36] Bosma H Marmot MG Hemingway H Nicholson AC Brunner E Stansfeld SA Low job control and risk of coronary heart disease in Whitehall II (prospective cohort) study Br Med J 1997 314 7080 558 565 10.1136/bmj.314.7080.558 PMC2126031 9055714

[B37] Karasek RA Job demands, job decision latitude, and mental strain: Implications for job redesign Adm Sci Q 1979 24 2 285 308 10.2307/2392498

[B38] Grzywacz JG Dooley D "Good jobs" to "bad jobs": replicated evidence of an employment continuum from two large surveys Soc Sci Med 2003 56 8 1749 1760 10.1016/S0277-9536(02)00170-3 12639591

[B39] Goldberg D Bridges K Duncan-Jones P Grayson D Detecting anxiety and depression in general medical settings Br Med J 1988 297 6653 897 899 10.1136/bmj.297.6653.897 PMC1834427 3140969

[B40] Slade T Johnston A Oakley Browne M Andrews G Whiteford H 2007 National Survey of Mental Health and Wellbeing: methods and key findings Aust N Z J Psychiatry 2009 43 7 594 605 10.1080/00048670902970882 19530016

[B41] Ware J Kosinski M Keller SD A 12-Item Short-Form Health Survey: construction of scales and preliminary tests of reliability and validity Med Care 1996 34 3 220 233 10.1097/00005650-199603000-00003 8628042

[B42] Carver CS White TL Behavioral inhibition, behavioral activation, and affective responses to impending reward and punishment: The BIS/BAS Scales J Pers Soc Psychol 1994 67 319 333 10.1037/0022-3514.67.2.319

[B43] Enders C A primer on maximum likelihood algorithms available for use with missing data Struct Equation Model 2001 8 128 141 10.1207/S15328007SEM0801_7

[B44] Dooley D Catalano R Economic change as a cause of behavioural disorder Psychol Bull 1980 87 450 468 10.1037/0033-2909.87.3.450 7384340

[B45] Winefield AH Cooper CL, Robertson IT Unemployment: Its psychological costs International Review of Industrial and Organizational Psychology 1995 London, Wiley

[B46] Hayes A Gray M Edwards B Social Inclusion: Origins Concepts and Key Themes Australian Institute of Family Studies, prepared for the Social Inclusion Unit, Department of the Prime Minister and Cabinet 2008 Australia

[B47] Average Weekly Earnings (Catalogue no. 6302.0) Australian Bureau of Statistics 2008 Australia

[B48] Labour Force Australia (Catalogue no. 6202.0) Australian Bureau of Statistics 2008 Australia

[B49] Census Tables - 2006 Census of Population and Housing (Catalogue no. 2068.0) Australian Bureau of Statistics 2006 Australia

[B50] Asch D Jedrziewski M Christakis N Response rates to mail surveys published in medical journals J Clin Epidemiol 1997 50 10 1129 1136 10.1016/S0895-4356(97)00126-1 9368521

[B51] Novo M Hammarström A Janlert U Does Low Willingness to Respond Introduce a Bias? Results from a Socio-epidemiological study among Young Men and Women Int J Soc Welfare 1999 8 2 155 163 10.1111/1468-2397.00076

[B52] Stansfeld S Clark C Caldwell T Rodgers B Power C Psychosocial work characteristics and anxiety and depressive disorders in midlife: The effects of prior psychological distress Occup Environ Med 2008 65 634 642 10.1136/oem.2007.036640 18388115

[B53] Agresti A An Introduction to Categorical Data Analysis 1996 New York. John Wiley and Sons, Inc 1996

